# (−)-Epigallocatechin 3-Gallate Synthetic Analogues Inhibit Fatty Acid Synthase and Show Anticancer Activity in Triple Negative Breast Cancer

**DOI:** 10.3390/molecules23051160

**Published:** 2018-05-11

**Authors:** Joan Crous-Masó, Sònia Palomeras, Joana Relat, Cristina Camó, Úrsula Martínez-Garza, Marta Planas, Lidia Feliu, Teresa Puig

**Affiliations:** 1 New Therapeutic Targets Laboratory (Targets Lab)-Oncology Unit, Department of Medical Sciences, University of Girona, Girona Institute for Biomedical Research, Emili Grahit 77, 17003 Girona, Spain; joan.crousmaso@gmail.com (J.C.-M.); sonia.palomeras@udg.edu (S.P.); 2 LIPPSO, Department of Chemistry, University of Girona, Maria Aurèlia Capmany 69, 17003 Girona, Spain; cristina.camo@udg.edu; 3 Department of Nutrition, Food Sciences and Gastronomy, School of Pharmacy and Food Sciences, Food and Nutrition Torribera Campus, University of Barcelona, Prat de la Riba 171, 08921 Santa Coloma de Gramenet, Spain; jrelat@ub.edu (J.R.); ursula-mtz@hotmail.com (Ú.M.-G.); 4 Institute of Nutrition and Food Safety of the University of Barcelona (INSA-UB), Prat de la Riba 171, 08921 Santa Coloma de Gramenet, Spain

**Keywords:** triple-negative breast cancer, fatty acid synthase, FASN inhibition, polyphenolic FASN inhibitors, (−)-epigallocatechin 3-gallate, synthetic analogues, apoptosis, anticancer activity

## Abstract

(−)-Epigallocatechin 3-gallate (EGCG) is a natural polyphenol from green tea with reported anticancer activity and capacity to inhibit the lipogenic enzyme fatty acid synthase (FASN), which is overexpressed in several human carcinomas. To improve the pharmacological profile of EGCG, we previously developed a family of EGCG derivatives and the lead compounds G28, G37 and G56 were characterized in HER2-positive breast cancer cells overexpressing FASN. Here, diesters G28, G37 and G56 and two G28 derivatives, monoesters M1 and M2, were synthesized and assessed in vitro for their cytotoxic, FASN inhibition and apoptotic activities in MDA-MB-231 triple-negative breast cancer (TNBC) cells. All compounds displayed moderate to high cytotoxicity and significantly blocked FASN activity, monoesters M1 and M2 being more potent inhibitors than diesters. Interestingly, G28, M1, and M2 also diminished FASN protein expression levels, but only monoesters M1 and M2 induced apoptosis. Our results indicate that FASN inhibition by such polyphenolic compounds could be a new strategy in TNBC treatment, and highlight the potential anticancer activities of monoesters. Thus, G28, G37, G56, and most importantly M1 and M2, are anticancer candidates (alone or in combination) to be further characterized in vitro and in vivo.

## 1. Introduction

Breast cancer is the most widespread cancer in women worldwide [1]. Although mortality has diminished in recent years owing to the programs of early diagnosis and to the improvement in treatment, this disease is still the first cause of death in women. Triple-negative breast cancer (TNBC) is a subtype of breast cancer in which the estrogen and the progesterone receptors are not expressed, and the human epidermal growth factor receptor 2 (HER2) is not amplified or overexpressed [2,3,4,5]. TNBC comprises about 15–20% of all breast cancers diagnosed. It tends to have an aggressive clinical course and to metastasize, resulting in early relapse and poor overall survival [2]. Patients with TNBC do not benefit from the targeted therapies used in other breast cancer subtypes, such as hormonal and anti-HER2 receptor therapies, thus leaving systemic cytotoxic chemotherapy as the sole treatment option [3,4,5]. The aggressiveness of this cancer and the scarcity of effective treatment options evidence the need of developing new therapeutic agents.

Metabolism deregulation is considered a hallmark of cancer [6,7]. In this sense, some metabolic enzymes such as fatty acid synthase (FASN) have been identified as valuable therapeutic targets for cancer treatment [8]. FASN is a homodimeric multienzymatic protein responsible for de novo fatty acid synthesis. It has a low or absent expression in normal tissues, but it is overexpressed and hyperactivated in many carcinomas such as breast cancer [8,9]. This overexpression correlates with a malignant phenotype and a poor disease prognosis. Therefore, the pharmacological inhibition of FASN is recognized as an attractive therapeutic approach. Blocking FASN activity triggers apoptosis in cancer cells and inhibits tumor growth in xenograft models [10], by disrupting lipid membrane synthesis, protein palmitoylation, and signalling of major oncogenic pathways [11,12]. Remarkably, FASN inhibition has minimal effect on non-malignant cells. Moreover, a recent study has shown that FASN is expressed in TNBC patient samples and that TNBC preclinical models benefit from FASN inhibition [13].

**Figure 1:**
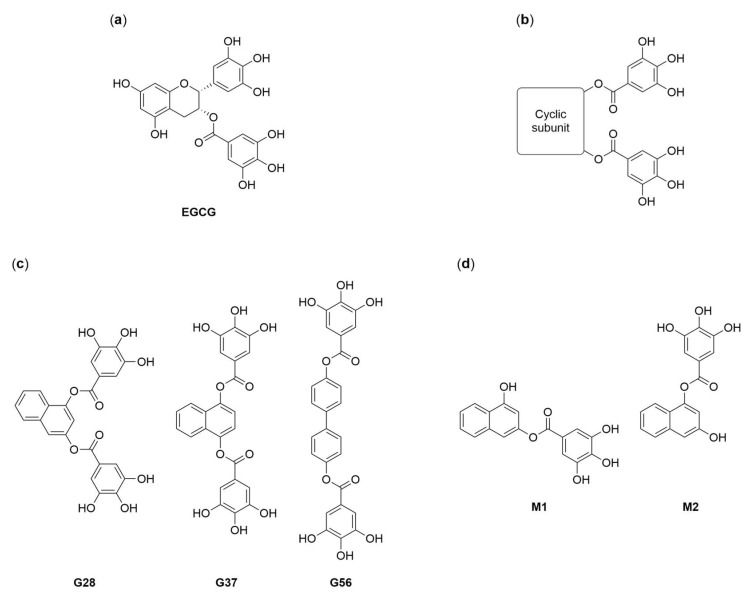
Structures of (−)-epigallocatechin 3-gallate (EGCG) (**a**), EGCG derivatives (**b**), the lead diesters (**c**) and the monoesters of G28 (**d**) [21,22,26].

The most representative FASN inhibitors are cerulenin, its synthetic derivative C75, and orlistat [9,11]. Even though these compounds have proven effective at controlling the progression of breast cancer, they have limitations that restrict their clinical development. These include chemical instability, low bioavailability, and stimulation of carnitine palmitoyltransferase-1 (CPT-1), which causes the acceleration of fatty acid β-oxidation and undesirable side effects such as body weight loss. On the other hand, epigallocatechin-3-gallate (EGCG, Figure 1a), the main polyphenolic catechin of green tea, has been described to inhibit FASN, to induce apoptosis in vitro and to reduce tumor size, without parallel CPT-1 stimulation or weight loss [9,13,14,15,16]. Specifically, the antiproliferative effects of EGCG have been widely reported in MDA-MB-231 TNBC cells [13,17,18,19], and such effects have also been associated, besides to FASN inhibition, to β-catenin downregulation [17,20]. Nevertheless, EGCG displays low potency, poor bioavailability, and limited stability in physiological conditions [21,22].

Considering this profile, EGCG’s structure inspired the design and synthesis of a novel collection of polyphenolic compounds, containing two galloyl moieties (3,4,5-trihydroxybenzoyl group) linked by a variable cyclic subunit (Figure 1b) [21,22]. The collection was screened for cytotoxicity and FASN inhibition against a HER2-positive human breast cancer cell line overexpressing FASN, and three lead compounds were identified, G28, G37, and G56 (Figure 1c), which improved the properties of their precursor molecule EGCG without affecting mice body weight [21,22,23]. G28 has been the most studied lead to date, proving effective in different HER2-positive breast cancer models, both in vivo and in vitro [10,24], and recently in TNBC in vitro models [25]. In addition, in vivo pharmacokinetic analyses have showed that G28 is hydrolyzed into two metabolites, M1 and M2 (Figure 1d) [23]. These compounds have already been tested for potential antibacterial activity [26] but, interestingly, not for anticancer activity. The purpose of the current study was to assess FASN inhibition by synthetic polyphenolic compounds as a potential therapeutic approximation for the clinically challenging TNBC. We synthesized diesters G28, G37 and G56 together with G28-derived monoesters M1 and M2, and we evaluated their cytotoxicity and capacity to inhibit FASN activity and to induce apoptosis in the MDA-MB-231 TNBC cell model.

## 2. Results

### 2.1. Synthesis of EGCG Analogues

The synthesis of diesters G28, G37 and G56 [21,22], and of monoesters M1 and M2 [26] was performed using procedures adapted from previously described protocols (Supplementary Materials) [23,26,27]. The synthesis encompassed the esterification of the corresponding aromatic diol with the conveniently protected acyl chloride derivative of gallic acid and the subsequent removal of the protecting groups. Diesters G28, G37 and G56 were obtained in 46, 65 and 61% overall yield, respectively, and monoesters M1 and M2 in 24 and 20% overall yield, respectively. All compounds were fully characterized by NMR and mass spectrometry. Additional NOESY and HMBC experiments allowed the unambiguous assignment of the regioisomeric monoesters M1 and M2.

### 2.2. Effect of EGCG Analogues on Cell Proliferation

The cytotoxic activity of G28, G37, G56 [21,22], M1 and M2 [26] was evaluated in the MDA-MB-231 human TNBC cell line. The IC_50_ values of compounds were determined from their dose-response curves (Table 1). This line has lower constitutive FASN expression levels than the SK-Br3 HER2-positive breast cancer cell line against which compounds were previously screened [21,22].

Diesters G28, G37 and G56 were more cytotoxic than their parent molecule EGCG (IC_50_ = 149 µM [13]), in particular being 1.9-, 1.4- and 3.3-fold more potent respectively. Monoesters M1 and M2 turned out to inhibit cell proliferation. In comparison to their parent compound G28 (77 µM), M2 exhibited the same cytotoxic activity (79 µM) and, interestingly, M1 doubled such cytotoxicity (41 µM).

### 2.3. Effect of EGCG Analogues on FASN Activity and on FASN Protein Expression

**Figure 2:**
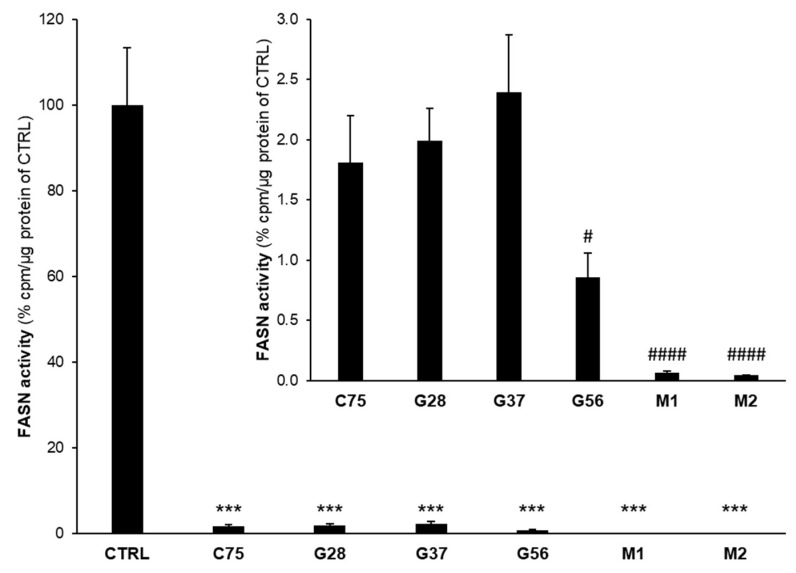
G28, G37, G56 [21,22], M1 and M2 [26] inhibit fatty acid synthase (FASN) activity in MDA-MB-231 TNBC cells. Cells were treated for 48 h with an IC_50_ concentration of G28, G37, G56, M1, M2, C75 or with dimethyl sulfoxide (DMSO). FASN activity was assayed by counting radiolabelled fatty acids synthesized de novo. Bars represent the remaining activity as percentage in treated cells versus untreated (control, CTRL) cells considered 100% activity. Data are mean ± SE from at least 4 assay points per condition in 3 independent experiments. ***, *p* < 0.001 versus control cells (DMSO). #, *p* < 0.05; ####, *p* < 0.001 versus C75-treated cells. Statistics were performed through Student’s *t*-test.

The capacity of G28, G37, G56 [21,22], M1 and M2 [26] to block FASN enzymatic activity in MDA-MB-231 cells was analyzed after a 48-h treatment with a concentration equal to the IC_50_ value of each compound. The inhibition is represented as the percentage of remaining activity with respect to untreated cells (Figure 2). The previously reported FASN-specific inhibitor C75 (IC_50_ = 46.6 ± 2.2 µM in MDA-MB-231 [13]) was used as a positive control.

Diesters G28, G37 and G56 significantly reduced FASN activity to 1.99 ± 0.27%, 2.39 ± 0.48%, and 0.86 ± 0.20% of untreated (control, CTRL) cells, respectively (all *p* = 0.000), to an equivalent extent as C75 (1.81 ± 0.39%). Monoesters M1 and M2, besides their cytotoxicity, were also able to reduce FASN activity in an even more powerful way than their parent G28. M1- and M2-treated cells displayed remaining FASN activities of 0.07 ± 0.01% and 0.04 ± 0.01%, respectively (both *p* = 0.000).

**Figure 3:**
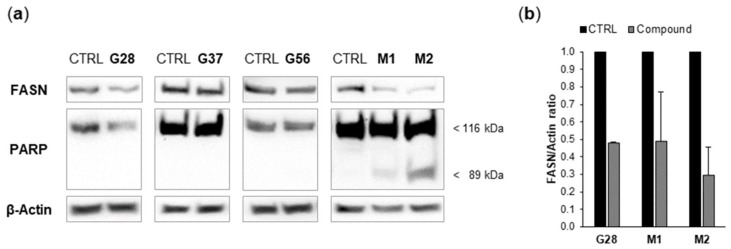
(**a**) G28, G37, G56 [21,22], M1 and M2 [26] differentially affect FASN protein expression in MDA-MB-231 TNBC cells, and only M1 and M2 induce apoptosis, as determined by poly(ADP-ribose) polymerase (PARP) cleavage. Cells were treated for 24 h with a 5-fold IC_50_ concentration of G28, G56, M1 and M2, a 2-fold IC_50_ concentration of G37, or with DMSO. Equal amounts of lysates were immunoblotted with anti-FASN and anti-PARP antibodies (the latter identified intact and cleaved PARP at 116 and 89 kDa, respectively). Blots were reproved for β-actin as loading control. Gels shown are equivalent to those obtained from two or three independent experiments. (**b**) G28, M1 and M2 downregulate FASN protein expression. FASN and β-actin immunoblot bands were quantified by densitometry and FASN/β-actin ratios of treatments are represented relative to those of controls. Data are mean ± SE from two or three independent experiments.

In parallel, in order to check whether the observed FASN activity decrease was related to changes in FASN protein levels, cells were treated with diesters and monoesters as described in the next Section 2.4 and FASN expression was analyzed by Western blot (Figure 3a). G37 and G56 did not modulate FASN protein levels, whereas G28 and its derivatives M1 and M2 displayed a tendency to reduce such levels (Figure 3b), which was more noticeable for M2.

### 2.4. Effect of EGCG Analogues on Apoptosis

Our group previously reported in SK-Br3 cells the activation of the apoptotic cell death mechanism by EGCG and its new-generation derivatives [14,15,16,21,22]. Accordingly, diesters [21,22] and monoesters [26] were evaluated in MDA-MB-231 cells for their ability to induce caspase activity, which causes cleavage of poly(ADP-ribose) polymerase (PARP). Cells were incubated for 24 h with concentrations corresponding to five-fold the IC_50_ values of G28, G56, M1 and M2, and two-fold the IC_50_ value of G37, because of the mortality induced by G37 at higher IC_50_ multiples (data not shown). PARP cleavage was analyzed by Western blot (Figure 3a). Apoptosis induction was not detected under the assayed conditions for any of the three diesters G28, G37 and G56. In contrast and interestingly, it was detected for both monoesters M1 and M2, with M2 showing a more intense band of cleaved PARP (89 kDa).

## 3. Discussion

TNBC is an aggressive cancer lacking a targeted therapy [3,4,5]. FASN is overexpressed in a variety of human carcinomas [8,9], including TNBC [28], and its inhibition with polyphenolic compounds such as EGCG has been demonstrated to be a promising therapeutic strategy, alone and in combination, for TNBC [13]. In this study, we characterized the anticancer effects of the EGCG analogues G28, G37 and G56 [21,22], and the G28 derivatives M1 and M2 [26], in MDA-MB-231 cells.

Firstly, the cytotoxic activity of compounds was determined. Diesters G28, G37 and G56 [21,22] displayed moderate IC_50_ values of 77, 104 and 45 µM, respectively, at 48-h exposure. Our group has studied the reference FASN inhibitors C75 and EGCG in several cancer cell lines, including MDA-MB-231 [13], in which the IC_50_ values were 46.6 ± 2.2 and 149.0 ± 6.7 µM, respectively. Thus, the three diesters are more potent than their parent molecule, as reported in SK-Br3 cells, and G56 is equally potent to C75. On the other hand, from the initial screening of EGCG analogues in SK-Br3 cells, some structure-activity relationships were inferred [22]. One of them was that cytotoxicity increases as the distance between the two galloyl moieties augments within the cyclic subunit. This could explain the superior cytotoxicity of G56, in which galloyl groups are in a relative 4,4′-position within a biphenyl system, further from each other than in the naphthalene ring of G28 and G37 (Figure 1c). Concerning monoesters M1 and M2 [26], which are metabolites of G28 in physiological conditions [23], they proved cytotoxic in MDA-MB-231 cells. M2 (IC_50_ = 79 µM) retained the activity of G28 while M1 (41 µM) enhanced that activity. This result raised the possibility that the in vivo antitumor efficacy of G28 is due, at least partially, to the two monoesters.

Beyond the obtained results, a positive correlation between the cytotoxic effects of the aforementioned inhibitors and cellular FASN expression has been reported [14,21,22,23], from the observation that compounds are less potent in MDA-MB-231 triple-negative cells, with low FASN levels, than in SK-Br3 HER2-positive cells, with high FASN levels. To support the correlation, it was demonstrated that the addition of exogenous palmitate, the end product of FASN, reduced the cytotoxicity of EGCG [14], and that the knockdown of FASN suppressed the cytotoxicity of G56 [22]. Hence, FASN inhibition plays a major role in the cytotoxicity of these polyphenolic compounds.

Secondly, the ability of the tested compounds [21,22,26] to block FASN activity was tackled. Compounds, at 48-h exposure, exerted a significant FASN activity reduction of 98.0%, 97.6% and 99.1% for diesters G28, G37 and G56, and of 99.93% and 99.96% for monoesters M1 and M2, respectively, as measured by an enzymatic radioassay. Moreover, G28 and its derivatives M1 and M2 also reduced FASN protein levels, unlike G37 and G56. Therefore, the observed FASN activity decrease in G28, M1 and M2 lysates could probably result not only from enzyme inhibition but also from diminished amounts of FASN protein. Our results show that, as for diesters [21,22], G56 is a more potent FASN inhibitor than G37 and G28, assuming that for G28 the remaining activity could be slightly higher in the absence of FASN downregulation (Figure 2). On the other hand, G28 stands out as the most effective diester due to its dual effect on FASN (activity and expression). Regarding monoesters [26], M1 and M2 affect FASN protein levels to a similar extent as G28 (Figure 3b) but, remarkably, both are better FASN inhibitors than G28 (Figure 2). These results indicate that the affinity for FASN of monoesters is greater than that of their parent molecule, being probably similar between the two monoesters.

In the original characterization, FASN inhibition values in SK-Br3 cells at 24-h exposure were 90 ± 4%, 69 ± 19% and 90 ± 5% for G28, G37 and G56, and 18% for EGCG, as measured by spectrophotometry, and FASN inhibition was not accompanied by downregulation of FASN expression [21,22]. These data evidence that diesters inhibit FASN more effectively than their parent molecule and agree with our results that G37 seems to be the less potent inhibitor. The observed FASN downregulation by G28 in MDA-MB-231 but not in SK-Br3 could be attributable to the concentration used, which was five-fold the IC_50_ value in our treatments against one-fold the IC_50_ value in the literature. Anyway, FASN downregulation has also been reported elsewhere, by C75 in A2780 ovarian cancer cells in a dose-dependent manner [29], and by EGCG, in A549 lung cancer cells [16] and in chemo-sensitive and -resistant MDA-MB-231 and HCC1806 TNBC cells [13]. A possible mechanism for this downregulation is that G28, apart from inhibiting FASN, could to some extent also inhibit the EGF receptor (EGFR), which is overexpressed in TNBC [30], thus impairing the downstream signalling that ends in FASN expression. This effect could occur at the transcriptional level, through PI3K and MAPK pathways activating the transcription factor SREBP-1c [31]; and at the translational level, through mTOR protein activating eIF4E and S6K [32]. Finally, FASN downregulation by monoesters M1 and M2 [26] is consistent with the result obtained for their parent molecule.

Beyond our results, in parallel to FASN inhibition and downregulation, EGCG and other polyphenols such as quercetin have also been described to reduce β-catenin protein expression [17,20,33,34]. β-catenin, when bound to E-cadherin complexes, functions as a cell-cell adhesion molecule, but when translocated to the nucleus acts as a key element in the Wnt signalling pathway, activating the transcription of target genes related to cell proliferation and metastasis, thus contributing to breast cancer progression [17,33]. Therefore, forthcoming studies in our group will investigate whether diesters and monoesters are able to modulate β-catenin expression and its nuclear accumulation in TNBC.

This is the first study testing monoesters M1 and M2 as potential anticancer agents. However, both monoesters have already been tested in another setting, as synthetic inhibitors of bacterial cell division targeting the GTP-binding site of protein FtsZ [26]. In that study, the affinity for FtsZ of monoesters, competing with GTP, was one order of magnitude lower than that of diester G28, with the affinity of M2 being slightly higher than that of M1, as determined by *K*_b_ comparison. The affinity of EGCG was two orders of magnitude lower relative to that of G28. Thus, only G28 was included in further biochemical, structural and antibacterial characterization. In striking contrast, our results suggested that the affinity for FASN is larger for monoesters than for G28. Aiming to understand this differential affinity, a future perspective of our group will be to gain insights, through molecular docking analyses, in the potential binding modes of G28 and monoesters to their target FASN catalytic domains. These domains are probably the same as those targeted by EGCG, the NADPH-dependent domains ketoreductase (KR) [35] and enoyl reductase (ER) [36,37,38,39], which participate in the saturation of the growing fatty acid chain [40]. Interestingly, EGCG has been shown to compete with NADPH/NADH for binding to KR and ER [35,36,37,38,39]. Since both NADPH/NADH (FASN) and GTP (FtsZ) are nucleotides, it is worth noting that, in the FtsZ study, the galloyl groups and the naphthalene scaffold of G28 were predicted to replace the interactions by the phosphates and the nucleobase of the nucleotide, respectively [26]. This could also be true in the interaction of G28 with FASN.

Thirdly, the capacity of compounds [21,22,26] to activate apoptotic cell death was addressed. The superior activity of monoesters was reaffirmed since, at 24-h exposure, monoesters, more importantly M2, induced PARP cleavage whereas diesters did not have this effect, despite using IC_50_ multiples as concentration. In the original characterization of diesters in SK-Br3 cells, an IC_50_ concentration value was sufficient to perceive truncated PARP as early as 12 h [21,22]. An equal result was obtained for C75 and EGCG in SK-Br3 and A549 cells [14,16], and for EGCG in chemo-sensitive and -resistant MDA-MB-231 and HCC1806 cells [13]. In addition, apoptosis has also been reported for EGCG combined with cetuximab in TNBC xenograft models [13]; and for G28, in other HER2-positive breast cancer models both in vivo and in vitro, alone and combined with anti-HER drugs [10], and in several ovarian cancer models in a dose-dependent manner [41,42].

Considering the effect of EGCG and diesters in SK-Br3, it is surprising that only EGCG shows effect in MDA-MB-231. The above described correlation between cytotoxicity and cellular FASN levels implies that MDA-MB-231 cells are less reliant on FASN activity than SK-Br3 cells for their survival and aggressiveness. Then, a possible hypothesis is that inhibition of FASN activity and expression by diesters in MDA-MB-231 may not be enough to compromise cell survival and reach the threshold for apoptosis induction, a threshold that may be comparatively lower in SK-Br3. In contrast, the two monoesters have larger affinities for FASN than those of diesters and both affect FASN expression, thus being able to overcome the threshold in MDA-MB-231. On the other hand, EGCG has been described to bind targets other than FASN [43]. Therefore, it may induce apoptosis through FASN inhibition along with other mechanisms, and so apoptosis may be detected irrespective of the cellular dependence on FASN.

In summary, in this study we evaluated the previously described G28 derivatives M1 and M2 [26] for first time as anticancer agents and partially disclosed their pharmacological profile in a TNBC model. Monoesters M1 and M2 turned out to be equally or more cytotoxic and to have a larger affinity for FASN than their parent molecule, to affect FASN expression and to induce apoptosis, which suggests that they could individually contribute to the in vivo antitumor activity of G28 after it has been hydrolyzed. In order to confirm this hypothesis, a future decisive study will be conducted to compare the efficacy of each monoester with that of G28 in a TNBC xenograft model. On the other hand, in the same in vitro model, the previously designed EGCG-derived diesters G28, G37 and G56 [21,22] exhibited cytotoxicity and remarkable FASN inhibition. Diesters, and especially monoesters, hold promise as target-directed anticancer drugs, either alone or in combination, focused on TNBC treatment. Furthermore, this work constitutes a starting point in our group for the future synthesis and evaluation of other polyphenolic compounds, such as the monoesters of G37 and G56, or computationally optimized compounds derived from the three diesters.

## 4. Materials and Methods 

### 4.1. Cell Culture

MDA-MB-231 breast carcinoma cells were obtained from the American Type Culture Collection (ATCC, Rockville, MD, USA). Cells were routinely grown in two-dimensional adherent conditions in Dulbecco’s Modified Eagle’s Medium (DMEM, Gibco, Waltham, MA, USA) supplemented with 10% heat-inactivated fetal bovine serum (FBS), 1% l-glutamine, 1% sodium pyruvate, 50 U/mL penicillin and 50 µg/mL streptomycin (HyClone Laboratories, Logan, UT, USA). Cells were maintained at 37 °C in a humidified atmosphere of 95% air and 5% CO_2_. The absence of *Mycoplasma* in cultures was checked before experiments.

### 4.2. Inhibition of Cell Proliferation

Dose-response experiments were done by means of the standard colorimetric MTT (3-4,5-dimethylthiazol-2-yl-2,5-diphenyltetrazolium bromide) reduction assay. Cells were plated out at a density of 4 × 10^3^ cells/well in 96-well microtiter plates and were incubated overnight for attachment. Then, cells were exposed for 48 h to fresh medium containing a range of concentrations of the corresponding EGCG derivative (10–140 µM) or were exposed to fresh medium alone. An influence of the vehicle dimethyl sulfoxide (DMSO) in the cytotoxicity of compounds was discarded. Following treatment, cells were incubated for 3 h with drug-free medium (100 µL/well) and 5 mg/mL MTT solution (Sigma-Aldrich, St. Louis, MO, USA; 10 µL/well). The formazan crystals formed by metabolically viable cells were solubilized in DMSO and their absorbance (Abs) was measured at 570 nm (Benchmark Plus microplate spectrophotometer, Bio-Rad Laboratories, Hercules, CA, USA). Using test (T) and control (C) average Abs values, the percentage of cell proliferation inhibition (CPI) at each concentration was calculated from the formula CPI = 100(1−T/C). By interpolation in the trendline of the resulting data points, the compound concentration that triggered 50% CPI (IC_50_ value) was determined. The assay was performed six times per compound.

### 4.3. Inhibition of Fatty Acid Synthase Activity

Cells were plated out at a density of 5 × 10^4^ cells/well in 24-well plates. Following overnight cell adherence, the medium was replaced by DMEM supplemented with 1% lipoprotein-deficient FBS (Sigma-Aldrich) along with the corresponding concentrations of compounds or DMSO. Treatments were maintained for 48 h, and for the last 6 h (1,2-^14^C) Acetic Acid Sodium salt (53.9 mCi/mmol, Perkin Elmer Biosciences, Waltham, MA, USA) was added to the medium (0.5 μCi/mL). The lipid extraction was done as previously described in [16]. Briefly, cells were harvested and washed twice with phosphate-buffered saline (PBS, HyClone Laboratories, Logan, UT, USA) and once with MeOH/PBS (2:3). Cells were then resuspended in 0.2 M NaCl and were lysed with freeze-thaw cycles. Lipids from cell debris were extracted with CHCl_3_/phenol (2:1) and 0.1 M KOH, and the organic phase was washed with CHCl_3_/MeOH/H_2_O (3:48:47). After solvents evaporation, pellets were resuspended in EtOH and transferred to a vial for radioactive counting. The total protein content in cell debris was quantified by the Bradford assay (Sigma-Aldrich).

### 4.4. Immunoblot Analysis of Cell Lysates

After a 24-h exposure to the corresponding EGCG derivative or DMSO, culture appearance was observed under microscope and cells were harvested by treatment with trypsin-ethylenediaminetetraacetic acid (EDTA) solution (Linus, Cultek, Madrid, Spain), together with supernatant cell debris. Cells plus debris were washed with PBS and lysed in ice-cold lysis buffer (Cell Signaling Technology, Danvers, MA, USA) supplemented with 2 mM phenylmethanesulfonyl fluoride (PMSF, Sigma-Aldrich) by vortexing every 5 min for 30 min. Particle-free lysates were obtained and their total protein content was determined by the Lowry-based DC Protein Assay (Bio-Rad Laboratories). Lysates’ equal protein amounts (30 µg) were heated in LDS Sample Buffer with Sample Reducing Agent (Invitrogen, Waltham, MA, USA) for 10 min at 70 °C, were run on sodium dodecyl sulphate polyacrylamide gel electrophoresis (SDS-PAGE, 7.5% polyacrylamide) and were transferred onto nitrocellulose membranes (Thermo Scientific, Pierce Protein Biology, Waltham, MA, USA). Blots were incubated for 1 h at room temperature in blocking buffer [5% powdered skimmed milk in tris-buffered saline with Tween 20 (TBST; 10 mM Tris-HCl pH 8.0, 150 mM NaCl, 0.1% Tween 20)] to avoid non-specific antibody binding. Then, blot fragments were incubated overnight at 4 °C with the appropriate primary antibody diluted in blocking buffer. Primary antibodies were rabbit polyclonal antibodies against FASN (Assay Designs, Ann Arbor, MI, USA; 905-069; dilution 1:1500) and PARP (Cell Signaling Technology; #9542; dilution 1:1000), and the mouse monoclonal antibody against β-actin (Cell Signaling Technology; #3700; dilution 1:2500). β-actin was used as a control of protein loading and transfer. Next, blots were washed in TBST and were incubated for 1 h at room temperature with the corresponding horseradish peroxidase (HRP)-conjugated goat secondary antibody diluted in blocking buffer, against rabbit (Cell Signaling Technology; #7074; dilution 1:4000) or mouse (Merck Millipore, Darmstadt, Germany; #401215; dilution 1:5000) antibodies. Lastly, blots were washed again and revealed (ChemiDoc MP Imaging System, Bio-Rad Laboratories) employing a chemiluminescent HRP substrate [Immobilon Western (Merck Millipore) or SuperSignal West Femto (Thermo Scientific, Pierce Protein Biology)]. The immunoblot analysis for each compound was repeated two or three times and a representative result is shown. FASN and β-actin bands were quantified using ImageJ software (version 1.51j8, National Institutes of Health, Bethesda, MD, USA).

### 4.5. Statistical Analysis

All data are expressed as mean ± standard error (SE). FASN inhibition data were analyzed by Student’s *t*-test and levels of statistical significance were *p*  <  0.001 (***) versus control cells, and *p*  <  0.05 (#), *p*  <  0.001 (####) versus C75-treated cells.

## Figures and Tables

**Table 1:** Cytotoxicity in MDA-MB-231 cancer cells of diesters and monoesters.

Compound	IC_50_ (µM) ^a^
**EGCG**	149.0 ± 6.7 ^b^
**G28** ^c^	77.3 ± 3.4
**G37** ^c^	103.7 ± 1.9
**G56** ^c^	45.4 ± 3.4
**M1** ^d^	41.4 ± 1.5
**M2** ^d^	78.9 ± 4.6

^a^ Data are mean ± SE from six independent experiments performed in triplicate. ^b^ Datum taken from [13]. ^c^ Compound described in [21,22]. ^d^ Compound described in [26].

## Supplementary Materials

The following are available online, Synthesis of EGCG analogues.
