# Supplementary material for: (−)-Epigallocatechin 3-Gallate Synthetic Analogues Inhibit Fatty Acid Synthase and Show Anticancer Activity in Triple Negative Breast Cancer
Source: Molecules. 2018 May 11;23(5):1160. doi: 10.3390/molecules23051160 (PMC6099607; doi:10.3390/molecules23051160)
Supplement: Supplementary file 1 [file molecules-23-01160-s001.pdf]

Supporting Information

(-)-Epigallocatechin 3-Gallate (EGCG) Synthetic Analogues Inhibit Fatty Acid Synthase and Show Anticancer Activity in Triple Negative Breast Cancer

Joan Crous-Masó, Sònia Palomeras, Joana Relat, Cristina Camó, Úrsula Martínez-Garza, Marta Planas*, Lidia Feliu* and Teresa Puig*

Table of Contents

Synthesis of EGCG Analogues

Synthesis of EGCG Analogues

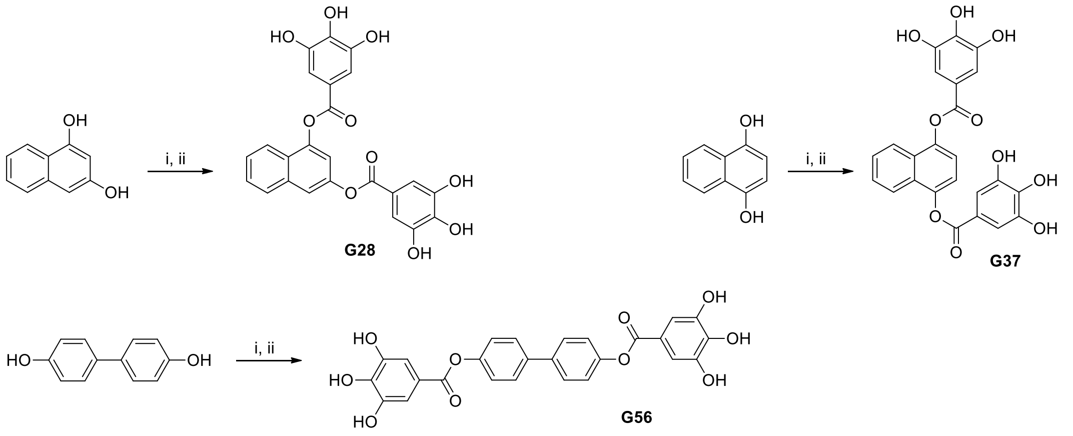

Scheme 1. Synthesis of diesters **G28**, **G37** and **G56**. Reagents and conditions: (i) 3,4,5-tris(*tert*-butyldimethylsilyloxy)benzoyl chloride (4 equiv.), Et_3N (4 equiv.), THF, rt, overnight. (ii) 1 M TBAF (6.5 or 7 equiv.), THF, rt, 15-20 min.

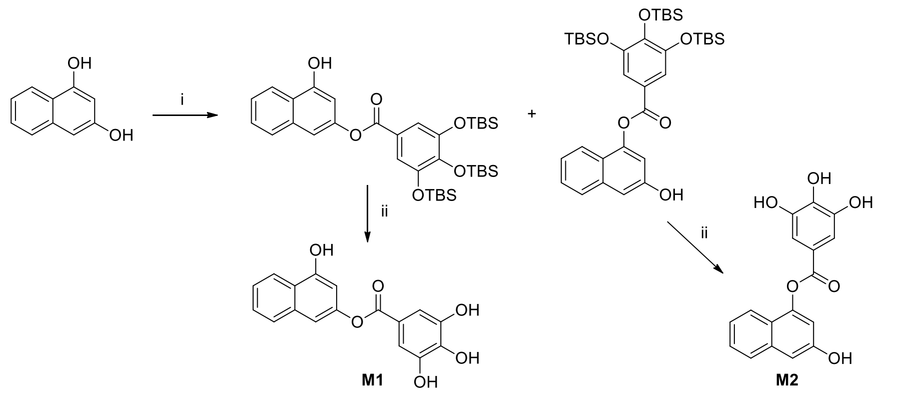

Scheme 2. Synthesis of monoesters **M1** and **M2**. Reagents and conditions: (i) 3,4,5-tris(*tert*-butyldimethylsilyloxy)benzoyl chloride (0.25 equiv.), Et_3N (1 equiv.), CH_2Cl_2, rt, overnight. (ii) 1 M TBAF (3.5 equiv.), THF, rt, 5 min.

1. Materials and Instruments

Reagents and solvents were purchased from commercial suppliers Sigma-Aldrich (St. Louis, MO, USA), Carlo Erba (Val de Reuil, France), Panreac (Castellar del Vallès, Spain) and Merck (Darmstadt, Germany), and were used without further purification.

Analytical thin layer chromatography (TLC) was performed on precoated TLC plates, silica gel 60 F_{254} (Merck). The spots on the TLC plates were visualized with UV/visible light (254 nm). Flash chromatography (FC) purifications were performed on silica gel 60 (0.040-0.063 mm, Merck).

^{1}H- and ^{13}C-NMR spectra were recorded on a Bruker Ultrashield Avance 300 or 400 MHz instrument (Serveis Tècnics de Recerca, University of Girona). Chemical shifts were reported as δ values (ppm) directly referenced to the solvent signal.

ESI-MS analyses were performed with an Esquire 6000 ESI Bruker ion Trap LC/MS instrument equipped with an electrospray ion source (Serveis Tècnics de Recerca, University of Girona). The instrument was operated in both positive ESI *m*/*z*(+) and negative ESI *m*/*z*(-) ion modes. Samples (5 μL) were introduced into the mass spectrometer ion source directly through a 1200 Series Agilent HPLC autosampler. The mobile phase, CH_3CN/H_2O (80:20), was delivered by an Agilent 1200 Series HPLC pump at a flow rate of 100 μL/min. Nitrogen was employed as both drying and nebulizing gas.

HRMS were recorded under conditions of ESI with a Bruker MicroTOF-Q II instrument using a hybrid quadrupole time-of-flight mass spectrometer (Serveis Tècnics de Recerca, University of Girona). Samples were introduced into the mass spectrometer ion source by direct infusion through a syringe pump and were externally calibrated using sodium formate. The instrument was operated in the positive ESI *m*/*z*(+) ion mode.

2. Synthesis of 3,4,5-tris(*tert*-butyldimethylsilyloxy)benzoic acid

N,N′-Diisopropylethylamine (12.2 mL, 69.8 mmol, 5.94 equiv.) was added to a solution of gallic acid (2.0 g, 11.8 mmol, 1 equiv.) and *tert*-butyldimethylsilyl chloride (8.9 g, 57.1 mmol, 4.86 equiv.) in anhydrous DMF. The mixture was stirred overnight at room temperature under nitrogen. The progress of the reaction was monitored by TLC. Once the reaction was completed, the mixture was neutralized with cold 1 M H_3PO_4 (18 mL) and extracted with hexane (3 × 60 mL). The combined organic layers were washed with saturated $NaHCO_3$ (2 × 60 mL) and brine (2 × 60 mL), dried over anhydrous Na_2SO_4 and filtered. The solvent was removed under reduced pressure affording a white solid. This solid was dissolved in H_2O/THF/acetic acid (1:1:3, 70 mL) and stirred at room temperature for 1 h. Then, cold H_2O was added, which was followed by THF removal under reduced pressure and extractions with diethyl ether/hexane (1:1, 2 × 140 mL). The combined organic layers were washed with H_2O (2 × 140 mL) and brine (2 × 140 mL), dried over anhydrous Na_2SO_4 and filtered. Removal of the solvent under reduced pressure, yielded 3,4,5-tris(*tert*-butyldimethylsilyloxy)benzoic acid as a white solid (4.5 g, 75% yield), which was used in the next step without purification. TLC (AcOEt/NH_3/MeOH, 6:2:1): R_f = 0.41. ^{1}H-NMR (400 MHz, $CDCl_3$) δ (ppm): 0.15 (s, 6 H, $(CH_3)_2$SiO *para*), 0.24 (s, 12 H, $(CH_3)_2$SiO *meta*), 0.95 (s, 18 H, $(CH_3)_3$CSiO *meta*), 0.99 (s, 9 H, $(CH_3)_3$CSiO *para*), 7.28 (s, 2 H, 2 CH_{gal}-3). ESI-MS (*m*/*z*) (+): 513.3 $[M+H]^+$. ESI-MS (*m*/*z*) (-): 511.2 $[M-H]^-$.

3. Synthesis of 1,3-bis[(3,4,5-tris(*tert*-butyldimethylsilyloxy)benzoyl)oxy] naphthalene

3,4,5-Tris(*tert*-butyldimethylsilyloxy)benzoic acid (500 mg, 0.97 mmol, 1 equiv.) was dissolved in anhydrous toluene (12 mL) under nitrogen at room temperature and anhydrous DMF (4 µL, 0.048 mmol, 0.05 equiv.) was added. The mixture was heated at 50 ºC and, afterwards, a solution of $(COCl)_2$ (150 µL, 1.75 mmol, 1.5 equiv.) in anhydrous toluene (1.4 mL) was added dropwise. After stirring the resulting solution at 50 º C for 1 h, the reaction was allowed to cool to room temperature and the solvent was removed under reduced pressure affording 3,4,5-tris(*tert*-butyldimethylsilyloxy)benzoyl chloride, which was immediately used without purification.

3,4,5-Tris(*tert*-butyldimethylsilyloxy)benzoyl chloride (4 equiv.) was dissolved in anhydrous THF (8 mL) under nitrogen at room temperature, and a solution of 1,3-dihydroxynaphtalene (39 mg, 0.24 mmol, 1 equiv.) and triethylamine (135 µL, 0.98 mmol, 4 equiv.) in anhydrous THF (1.4 mL) was added dropwise. The resulting mixture was stirred overnight at room temperature under nitrogen. The progress of the reaction was monitored by TLC. Once the reaction was completed, the solvent was removed under reduced pressure and the resulting orange residue was dissolved in CH_2Cl_2, washed with saturated $NaHCO_3$ (3 × 30 mL) and H_2O (3 × 30 mL). Then, the organic layer was dried over anhydrous Na_2SO_4, filtered and the solvent was removed under reduced pressure. Purification of the resulting orange solid by flash chromatography using hexane/AcOEt (9:1) yielded 1,3-bis[(3,4,5-tris(*tert*-butyldimethylsilyloxy)benzoyl)oxy]naphthalene as an oil (198 mg, 73% yield). TLC (Hexane/AcOEt, 3:1): R_f = 0.76. ^{1}H-NMR (400 MHz, $CDCl_3$) δ (ppm): 0.18 (s, 6 H, $(CH_3)_2$SiO *para*), 0.20 (s, 6 H, $(CH_3)_2$SiO *para*), 0.27 (s, 12 H, $(CH_3)_2$SiO *meta*), 0.28 (s, 12 H, $(CH_3)_2$SiO *meta*), 0.97 (s, 18 H, $(CH_3)_3$CSiO *meta*), 0.98 (s, 18 H, $(CH_3)_3$CSiO *meta*), 1.01 (s, 9 H, $(CH_3)_3$CSiO *para*), 1.03 (s, 9 H, $(CH_3)_3$CSiO *para*), 7.38 (d, *J* = 1.8 Hz, 1 H, CH_{naph}-6), 7.40 (s, 2 H, CH_{gal}-3), 7.46-7.55 (m, 4 H, CH_{gal}-3, CH_{naph}-11,12), 7.61 (d, *J* = 1.8 Hz, 1 H, CH_{naph}-8), 7.86 (d, *J* = 7.8 Hz, 1 H, CH_{naph}-10), 7.98 (d, *J* = 7.8 Hz, 1 H, CH_{naph}-13). ^{13}C-NMR (100 MHz, $CDCl_3$) δ (ppm): -3.6 (4 CH_3, $(CH_3)_2$SiO *para*), -3.3 (8 CH_3, $(CH_3)_2$SiO *meta*), 18.5 (2 C, $(CH_3)_3$CSiO *para*), 18.8 (4 C, $(CH_3)_3$CSiO *meta*), 26.0 (6 CH_3, $(CH_3)_3$CSiO *para*), 26.1 (12 CH_3, $(CH_3)_3$CSiO *meta*), 114.4 (CH_{naph}-6 or CH_{naph}-8), 116.2 (4 CH_{gal}-3), 116.6 (CH_{naph}-6 or CH_{naph}-8), 120.7, 121.0 (2 C_{gal}-4), 121.5, 125.3, 126.0, 127.1, 127.8 (C_{naph}-14, CH_{naph}-10,11,12,13), 134.3 (C_{naph}-9), 144.1, 144.2 (2 C_{gal}-1), 147.5, 148.1 (C_{naph}-5,7), 148.7, 148.8 (4 C_{gal}-2), 164.4, 164.8 (2 C=O). ESI-MS (*m*/*z*): 1171.1 $[M+Na]^+$.

4. Synthesis of 1,4-bis[(3,4,5-tris(*tert*-butyldimethylsilyloxy)benzoyl)oxy] naphthalene

This compound was prepared following the same procedure described for 1,3-bis[(3,4,5-tris(*tert*-butyldimethylsilyloxy)benzoyl)oxy]naphthalene but using 1,4-dihydroxynaphtalene. Final purification with hexane/CH_2Cl_2 (70:30) afforded 1,4-bis[(3,4,5-tris(*tert*-butyldimethylsilyloxy)benzoyl)oxy]naphthalene as a yellow solid (70% yield). TLC (Hexane/AcOEt, 4:1): R_f = 0.81. ^{1}H-NMR (400 MHz, $CDCl_3$) δ (ppm): 0.22 (s, 12 H, $(CH_3)_2$SiO *para*), 0.31 (s, 24 H, $(CH_3)_2$SiO *meta*), 1.00 (s, 36 H, $(CH_3)_3$CSiO *meta*), 1.05 (s, 18 H, $(CH_3)_3$CSiO *para*), 7.45 (s, 2 H, CH_{naph}-6), 7.53 (s, 4 H, CH_{gal}-3), 7.54-7.57 (m, 2 H, CH_{naph}-9), 8.00-8.03 (m, 2 H, CH_{naph}-8). ^{13}C-NMR (100 MHz, $CDCl_3$) δ (ppm): -3.7 (4 CH_3, $(CH_3)_2$SiO *para*), -3.4 (8 CH_3, $(CH_3)_2$SiO *meta*), 18.7 (2 C, $(CH_3)_3$CSiO *para*), 19.0

(4 C, $(CH_3)_3CSiO$ *meta*), 26.3 (6 CH_3, $(CH_3)_3CSiO$ *para*), 26.4 (12 CH_3, $(CH_3)_3CSiO$ *meta*), 116.3 (4 CH_{gal}-3), 118.0 (2 CH_{naph}-6), 121.1 (2 C_{gal}-4 or 2 C_{naph}-7), 121.9 (2 CH_{naph}-8), 127.0 (2 CH_{naph}-9), 128.1 (2 C_{gal}-4 or 2 C_{naph}-7), 144.3, 144.7 (2 C_{gal}-1, 2 C_{naph}-5), 148.9 (4 C_{gal}-2), 164.8 (2 C=O).

5. Synthesis of 4,4′-bis[(3,4,5-tris(*tert*-butyldimethylsilyloxy)benzoyl)oxy]-1,1′-biphenyl

This compound was prepared following the same procedure described for 1,3-bis[(3,4,5-tris(*tert*-butyldimethylsilyloxy)benzoyl)oxy]naphthalene but using 14,4′-dihydroxy-1,1′-biphenyl. Final purification with hexane/CH_2Cl_2 (70:30) afforded 4,4′-bis[(3,4,5-tris(*tert*-butyldimethylsilyloxy)benzoyl)oxy]-1,1′-biphenyl as a white solid (71% yield). TLC (Hexane/AcOEt, 4:1): R_f = 0.81. ^{1}H-NMR (400 MHz, $CDCl_3$) δ (ppm): 0.19 (s, 12 H, $(CH_3)_2SiO$ *para*), 0.28 (s, 24 H, $(CH_3)_2SiO$ *meta*), 0.99 (s, 36 H, $(CH_3)_3CSiO$ *meta*), 1.03 (s, 18 H, $(CH_3)_3CSiO$ *para*), 7.30 (d, *J* = 8.8 Hz, 4 H, CH_{biph}-6), 7.41 (s, 4 H, CH_{gal}-3), 7.63 (d, *J* = 8.8 Hz, 4 H, CH_{biph}-7). ^{13}C-NMR (100 MHz, $CDCl_3$) δ (ppm): -3.7 (4 CH_3, $(CH_3)_2SiO$ *para*), -3.5 (8 CH_3, $(CH_3)_2SiO$ *meta*), 18.7 (2 C, $(CH_3)_3CSiO$ *para*), 19.0 (4 C, $(CH_3)_3CSiO$ *meta*), 26.3 (6 CH_3, $(CH_3)_3CSiO$ *para*), 26.4 (12 CH_3, $(CH_3)_3CSiO$ *meta*), 116.2 (4 CH_{gal}-3), 121.3 (2 C_{gal}-4), 122.2 (4 CH_{biph}-6), 128.3 (4 CH_{biph}-7), 138.3 (2 C_{biph}-8), 144.1 (2 C_{gal}-1 or 2 C_{biph}-5), 148.8 (4 C_{gal}-2), 150.8 (2 C_{gal}-1 or 2 C_{biph}-5), 165.0 (2 C=O).

6. Synthesis of 4-hydroxy-2-naphthyl 3,4,5-tris(*tert*-butyldimethylsilyloxy)benzoate and 3-hydroxy-1-naphthyl 3,4,5-tris(*tert*-butyldimethylsilyloxy)benzoate

3,4,5-Tris(*tert*-butyldimethylsilyloxy)benzoic acid (1.312 g, 2.56 mmol, 1 equiv.) was dissolved in anhydrous toluene (20 mL) under nitrogen at room temperature and anhydrous DMF (20 μL, 0.26 mmol, 0.1 equiv.) was added. The mixture was heated at 50 ºC and, afterwards, a solution of $(COCl)_2$ (330 μL, 3.84 mmol, 1.5 equiv.) in anhydrous toluene (2 mL) was added dropwise. After stirring the resulting solution at 50 º C for 1 h, the reaction was allowed to cool to room temperature and the solvent was removed under reduced pressure affording 3,4,5-tris(*tert*-butyldimethylsilyloxy)benzoyl chloride, which was immediately used without purification.

A solution of 3,4,5-tris(*tert*-butyldimethylsilyloxy)benzoyl chloride (1 equiv.) in anhydrous CH_2Cl_2 (10 mL) was added dropwise to a solution of 1,3-dihydroxynaphtalene (1.655 g, 10.23 mmol, 4 equiv.) and triethylamine (1.43 mL, 10.23 mmol, 4 equiv.) in anhydrous CH_2Cl_2 (10 mL). The resulting dark red mixture was stirred overnight at room temperature under nitrogen. The progress of the reaction was monitored by TLC. Once the reaction was completed, the crude was washed with saturated $NaHCO_3$ (3 × 30 mL) and H_2O (3 × 45 mL). Then, the organic layer was dried over anhydrous Na_2SO_4, filtered and the solvent was removed under reduced pressure. The resulting brown oil was purified by flash chromatography using mixtures of hexane/CH_2Cl_2 of increasing polarity. Elution with hexane/CH_2Cl_2 (40:60) yielded 4-hydroxy-2-naphthyl 3,4,5-tris(*tert*-butyldimethylsilyloxy)benzoate as an orange solid (478 mg, 29% yield) and elution with hexane/CH_2Cl_2 from (30:70) to (10:90) afforded 3-hydroxy-1-naphthyl 3,4,5-tris(*tert*-butyldimethylsilyloxy)benzoate as a brown solid (508 mg, 30% yield).

4-Hydroxy-2-naphthyl 3,4,5-tris(tert-butyldimethylsilyloxy)benzoate. TLC (Hexane/AcOEt, 3:1): R_f = 0.54. ^{1}H-NMR (400 MHz, $CDCl_3$) δ (ppm): 0.20 (s, 6 H, $(CH_3)_2$SiO *para*), 0.29 (s, 12 H, $(CH_3)_2$SiO *meta*), 0.99 (s, 18 H, $(CH_3)_3$CSiO *meta*), 1.03 (s, 9 H, $(CH_3)_3$CSiO *para*), 6.73 (d, *J* = 2.2 Hz, 1 H, CH_{naph}-6), 6.82 (br, 1 H, OH), 7.22 (d, *J* = 2.2 Hz, 1 H, CH_{naph}-8), 7.33 (td, *J* = 8.2 Hz, *J'* = 1.0 Hz, 1 H, CH_{naph}-12), 7.44 (s, 2H, 2 CH_{gal}-3), 7.45 (td, *J* = 8.2 Hz, *J'* = 1.0 Hz, 1 H, CH_{naph}-11), 7.74 (d, *J* = 8.2 Hz, 1 H, CH_{naph}-10), 7.96 (d, *J* = 8.2 Hz, 1 H, CH_{naph}-13). ^{13}C-NMR (100 MHz, $CDCl_3$) δ (ppm): -3.8 (2 CH_3, $(CH_3)_2$SiO *para*), -3.6 (4 CH_3, $(CH_3)_2$SiO *meta*), 18.5 (C, $(CH_3)_3$CSiO *para*), 18.8 (2 C, $(CH_3)_3$CSiO *meta*), 26.1 (3 CH_3, $(CH_3)_3$CSiO *para*), 26.2 (6 CH_3, $(CH_3)_3$CSiO *meta*), 104.5 (CH_{naph}-6), 110.6 (CH_{naph}-8), 116.2 (2 CH_{gal}-3), 121.0, 122.0, 123.0, 124.7 (C_{naph}-14, CH_{naph}-12,13, C_{gal}-4), 127.0, 127.3 (CH_{naph}-10,11), 134.5 (C_{naph}-9), 144.3 (C_{gal}-1), 148.6 (C_{naph}-7), 148.7 (2 C_{gal}-2), 153.1 (C_{naph}-5), 165.9 (C=O). ESI-MS (*m/z*) (+): 655.3 $[M+H]^+$, 677.4 $[M+Na]^+$. ESI-MS (*m/z*) (-): 539.1 $[M-H]^-$, 653.3 $[M-H]^-$.

3-Hydroxy-1-naphthyl 3,4,5-tris(tert-butyldimethylsilyloxy)benzoate. TLC (Hexane/AcOEt, 3:1): R_f = 0.40. ^{1}H-NMR (400 MHz, $CDCl_3$) δ (ppm): 0.20 (s, 6 H, $(CH_3)_2$SiO *para*), 0.28 (s, 12 H, $(CH_3)_2$SiO *meta*), 0.98 (s, 18 H, $(CH_3)_3$CSiO *meta*), 1.03 (s, 9 H, $(CH_3)_3$CSiO *para*), 5.37 (s, 1 H, OH), 7.04 (d, *J* = 2.4 Hz, 1 H, CH_{naph}-6), 7.09 (d, *J* = 2.4 Hz, 1 H, CH_{naph}-8), 7.33 (td, *J* = 8.3 Hz, *J'* = 1.2 Hz, 1 H, CH_{naph}-12), 7.44 (td, *J* = 8.3 Hz, *J'* = 1.2 Hz, 1 H, CH_{naph}-11), 7.49 (s, 2 H, 2 CH_{gal}-3), 7.66 (d, *J* = 8.3 Hz, 1 H, CH_{naph}-10), 7.86 (d, *J* = 8.3 Hz, 1 H, CH_{naph}-13). ^{13}C-NMR (100 MHz, $CDCl_3$) δ (ppm): -3.8 (2 CH_3, $(CH_3)_2$SiO *para*), -3.6 (4 CH_3, $(CH_3)_2$SiO *meta*), 18.5 (C, $(CH_3)_3$CSiO *para*), 18.8 (2 C, $(CH_3)_3$CSiO *meta*), 26.1 (3 CH_3, $(CH_3)_3$CSiO *para*), 26.2 (6 CH_3, $(CH_3)_3$CSiO *meta*), 107.8 (CH_{naph}-8), 111.0 (CH_{naph}-6), 116.2 (2 CH_{gal}-3), 120.6, 121.1, 122.3, 123.8 (C_{naph}-14, CH_{naph}-12,13, C_{gal}-4), 126.6, 126.9 (CH_{naph}-10,11), 135.2 (C_{naph}-9), 144.3 (C_{gal}-1), 147.6 (C_{naph}-5), 148.8 (2 C_{gal}-2), 153.3 (C_{naph}-7), 165.1 (C=O). ESI-MS (*m/z*) (+): 655.3 $[M+H]^+$, 677.3 $[M+Na]^+$. ESI-MS (*m/z*) (-): 539.2 $[M-H]^-$, 653.2 $[M-H]^-$.

7. Synthesis of 1,3-bis[(3,4,5-trihydroxybenzoyl)oxy)]naphthalene (G28)

A solution of 1 M TBAF (1.02 mL, 1.02 mmol, 6.5 equiv.) was added to a solution of 1,3-bis[(3,4,5-tris(*tert*-butyldimethylsilyloxy)benzoyl)oxy]naphthalene (181 mg, 0.16 mmol, 1 equiv.) in anhydrous THF (12 mL) under nitrogen. The reaction mixture was stirred under nitrogen at room temperature for 15 min. The progress of the reaction was monitored by TLC. Once the reaction was completed, cold 1 M H_3PO_4 (10 mL) was added and the product was extracted with diethyl ether. The combined organic layers were washed with brine, dried over anhydrous Na_2SO_4 and filtered. The solvent was evaporated under reduced pressure and the resulting orange oil was purified by flash chromatography using mixtures of CH_2Cl_2/MeOH of increasing polarity. Elution with CH_2Cl_2/MeOH (95:5) afforded 1,3-bis[(3,4,5-trihydroxybenzoyl)oxy)]naphthalene (**G28**) as a solid (47 mg, 63% yield). ^{1}H-NMR (400 MHz, CD_3OD) δ (ppm): 7.28 (s, 2 H, CH_{gal}-3), 7.29 (d, *J* = 2.2 Hz, 1 H, CH_{naph}-6), 7.36 (s, 2 H, CH_{gal}-3), 7.54 (td, *J* = 6.8 Hz, *J'* = 1.6 Hz, 1 H, CH_{naph}-11 or 12), 7.60 (td, *J* = 6.8 Hz, *J'* = 1.6 Hz, 1 H, CH_{naph}-11 or 12), 7.67 (d, *J* = 2.2 Hz, 1 H, CH_{naph}-8), 7.92 (d, *J* = 8.8 Hz, 1 H, CH_{naph}-10), 7.97 (d, *J* = 8.8 Hz, 1 H, CH_{naph}-13). ESI-MS (*m/z*): 462.9 $[M-H]^-$. HRMS (ESI) *m/z* (-): calculated for $C_{24}H_{14}O_{10}$ $[M-2H]^{2-}$ 231.0288, found 231.0282; calculated for $C_{24}H_{15}O_{10}$ $[M-H]^-$ 463.0660, found 463.0663.

8. Synthesis of 1,4-bis[(3,4,5-trihydroxybenzoyl)oxy]naphthalene (G37)

A solution of 1 M TBAF (1.24 mL, 1.24 mmol, 7 equiv.) was added to a solution of 1,4-bis[(3,4,5-tris(*tert*-butyldimethylsilyloxy)benzoyl)oxy]naphthalene (204 mg, 0.18 mmol, 1 equiv.) in anhydrous THF (15 mL) under nitrogen. The reaction mixture was stirred under nitrogen at room temperature for 20 min. The progress of the reaction was monitored by TLC. Once the reaction was completed, it was acidified to pH = 5 with cold 1 M H_3PO_4 (10 mL) and the solvents were removed under reduced pressure. The resulting brown solid was dried and purified by flash chromatography using mixtures of CH_2Cl_2/MeOH of increasing polarity. Elution with CH_2Cl_2/MeOH (95:5) and with CH_2Cl_2/MeOH/NH_3 (94.5:5:0.5) afforded 1,4-bis[(3,4,5-trihydroxybenzoyl)oxy]naphthalene (**G37**) as a brown solid (76 mg, 93% yield). TLC (CH_2Cl_2/MeOH, 17:3): R_f = 0.28. ^{1}H-NMR (400 MHz, CD_3OD) δ (ppm): 7.35 (s, 4 H, CH_{gal}-3), 7.38 (s, 2 H, CH_{naph}-6), 7.57-7.59 (m, 2 H, CH_{naph}-9), 7.92-7.94 (m, 2 H, CH_{naph}-8). ^{13}C-NMR (100 MHz, CD_3OD) δ (ppm): 110.7 (4 CH_{gal}-3), 119.2 (2 CH_{naph}-6), 120.2 (C_{gal}-4 or C_{naph}-7), 122.8 (2 CH_{naph}-8), 128.1 (2 CH_{naph}-9), 129.3 (C_{gal}-4 or C_{naph}-7), 140.9, 146.1 (2 C_{gal}-1, 2 C_{naph}-5), 146.9 (4 C_{gal}-2), 167.0 (2 C=O). HRMS (ESI) *m/z* (-): calculated for $C_{24}H_{14}O_{10}$ $[M-2H]^{2-}$ 231.0288, found 231.0292; calculated for $C_{24}H_{15}O_{10}$ $[M-H]^-$ 463.0660, found 463.0653.

9. Synthesis of 4,4′-bis[(3,4,5-trihydroxybenzoyl)oxy]-1,1′-biphenyl (G56)

A solution of 1 M TBAF (5.20 mL, 5.20 mmol, 7 equiv.) was added to a solution of 4,4′-bis[(3,4,5-tris(*tert*-butyldimethylsilyloxy)benzoyl)oxy]-1,1′-biphenyl (874 mg, 0.74 mmol, 1 equiv.) in anhydrous THF (15 mL) under nitrogen. The reaction mixture was stirred under nitrogen at room temperature for 20 min. The progress of the reaction was monitored by TLC. Once the reaction was completed, it was acidified to pH = 5 with cold 1 M H_3PO_4 (10 mL) and the solvents were removed under reduced pressure. The resulting orange solid was dried and purified by flash chromatography using mixtures of CH_2Cl_2/MeOH of increasing polarity. Elution with CH_2Cl_2/MeOH (95:5) and with CH_2Cl_2/MeOH/NH_3 (94.5:5:0.5) afforded 4,4′-bis[(3,4,5-trihydroxybenzoyl)oxy]-1,1′-biphenyl (**G56**) as a grey oil (312 mg, 86% yield). TLC (CH_2Cl_2/MeOH, 17:3): R_f = 0.34. ^{1}H-NMR (400 MHz, CD_3OD) δ (ppm): 7.23 (s, 4 H, CH_{gal}-3), 7.26 (d, *J* = 8.6 Hz, 4 H, CH_{biph}-6), 7.70 (d, *J* = 8.6 Hz, 4 H, CH_{biph}-7). ^{13}C-NMR (100 MHz, CD_3OD) δ (ppm): 110.6 (4 CH_{gal}-3), 120.5 (2 C_{gal}-4), 123.3 (4 CH_{biph}-6), 129.0 (4 CH_{biph}-7), 139.2 (2 C_{biph}-8), 140.6 (2 C_{gal}-1 or 2 C_{biph}-5), 146.7 (4 C_{gal}-2), 152.1 (2 C_{gal}-1 or 2 C_{biph}-5), 167.1 (2 C=O). HRMS (ESI) *m/z* (-): calculated for $C_{26}H_{16}O_{10}$ $[M-2H]^{2-}$ 244.0366, found 244.0365; calculated for $C_{26}H_{17}O_{10}$ $[M-H]^-$ 489.0816, found 489.0793.

10. Synthesis of 4-hydroxy-2-naphthyl 3,4,5-trihydroxybenzoate (M1)

A solution of 1 M TBAF (4.36 mL, 4.36 mmol, 3.5 equiv.) was added to a solution of 4-hydroxy-2-naphthyl 3,4,5-tris(*tert*-butyldimethylsilyloxy)benzoate (816 g, 1.25 mmol, 1 equiv.) in anhydrous THF (15 mL) under nitrogen. The reaction mixture was stirred under nitrogen at room temperature for 5 min. The progress of the reaction was monitored by TLC. Once the reaction was completed, it was acidified to pH = 6 with cold 1 M H_3PO_4 (5 mL) and THF was removed under reduced pressure. Afterwards, the product was extracted with diethyl ether (3 × 20 mL), the combined organic layers were washed with brine (2 × 60 mL), dried over anhydrous Na_2SO_4 and filtered. The solvent

was removed under reduced pressure and the resulting brown oil was purified by flash chromatography using mixtures of hexane/AcOEt of increasing polarity, from hexane/AcOEt (70:30) to AcOEt/MeOH/NH_3 (94.5:5:0.5), affording 4-hydroxy-2-naphthyl 3,4,5-trihydroxybenzoate (**M1**) as a green solid (320 mg, 82% yield). ^{1}H-NMR (400 MHz, CD_3COCD_3) δ (ppm): 6.84 (d, *J* = 2.0 Hz, 1 H, CH_{naph}-6), 7.24 (d, *J* = 2.0 Hz, 1 H, CH_{naph}-8), 7.31 (s, 2 H, 2 CH_{gal}-3), 7.45 (td, *J* = 7.6 Hz, *J'* = 1.2 Hz, 1 H, CH_{naph}-12), 7.51 (td, *J* = 7.6 Hz, *J'* = 1.2 Hz, 1 H, CH_{naph}-11), 7.83 (d, *J* = 8.4 Hz, 1 H, CH_{naph}-10), 8.24 (d, *J* = 8.4 Hz, 1 H, CH_{naph}-13). ^{13}C-NMR (100 MHz, CD_3COCD_3) δ (ppm): 105.0 (CH_{naph}-6), 110.4 (2 CH_{gal}-3), 110.8 (CH_{naph}-8), 121.0 (C_{naph}-14), 123.1 (CH_{naph}-13), 124.0 (C_{gal}-4), 125.2 (CH_{naph}-12), 127.8 (CH_{naph}-11), 128.2 (CH_{naph}-10), 135.6 (C_{naph}-9), 139.5 (C_{gal}-1), 146.3 (2 C_{gal}-2), 150.3 (C_{naph}-7), 155.0 (C_{naph}-5), 165.5 (C=O). ESI-MS (*m*/*z*) (+): 335.0 $[M+Na]^+$. ESI-MS (*m*/*z*) (-): 310.9 $[M-H]^-$. HRMS (ESI) *m*/*z* (-): calculated for $C_{17}H_{11}O_6$ $[M-H]^-$ 311.0550, found 311.0545.

11. Synthesis of 3-hydroxy-1-naphthyl 3,4,5-trihydroxybenzoate (M2)

A solution of 1 M TBAF (5.85 mL, 5.85 mmol, 3.5 equiv.) was added to a solution of 3-hydroxy-1-naphthyl 3,4,5-tris(*tert*-butyldimethylsilyloxy)benzoate (1.094 g, 1.67 mmol, 1 equiv.) in anhydrous THF (15 mL) under nitrogen. The reaction mixture was stirred under nitrogen at room temperature for 5 min. The progress of the reaction was monitored by TLC. Once the reaction was completed, it was acidified to pH = 6 with cold 1 M H_3PO_4 (5 mL) and the solvents were removed under reduced pressure. The resulting orange solid was dried and purified by flash chromatography using mixtures of hexane/AcOEt of increasing polarity, from hexane/AcOEt (60:40) to AcOEt/MeOH/NH_3 (94.5:5:0.5), affording 3-hydroxy-1-naphthyl 3,4,5-trihydroxybenzoate (**M2**) as a brown solid (356 mg, 68% yield). ^{1}H-NMR (400 MHz, CD_3COCD_3) δ (ppm): 7.09 (d, *J* = 2.4 Hz, 1 H, CH_{naph}-6), 7.17 (d, *J* = 2.4 Hz, 1 H, CH_{naph}-8), 7.31 (td, *J* = 7.6 Hz, *J'* = 1.2 Hz, 1 H, CH_{naph}-12), 7.40 (s, 2 H, 2 CH_{gal}-3), 7.43 (td, *J* = 7.6 Hz, *J'* = 1.2 Hz, 1 H, CH_{naph}-11), 7.75 (d, *J* = 8.4 Hz, 1 H, CH_{naph}-10), 7.79 (d, *J* = 8.4 Hz, 1 H, CH_{naph}-13). ^{13}C-NMR (100 MHz, CD_3COCD_3) δ (ppm): 107.9 (CH_{naph}-8), 110.5 (2 CH_{gal}-3), 112.3 (CH_{naph}-6), 120.5 (C_{naph}-14), 122.1 (CH_{naph}-13), 123.1 (C_{gal}-4), 124.2 (CH_{naph}-12), 127.3 (CH_{naph}-10), 127.7 (CH_{naph}-11), 136.5 (C_{naph}-9), 139.8 (C_{gal}-1), 146.4 (2 C_{gal}-2), 149.1 (C_{naph}-5), 155.9 (C_{naph}-7), 165.3 (C=O). ESI-MS (*m/z*) (+): 335.0 $[M+Na]^+$. ESI-MS (*m/z*) (-): 310.9 $[M-H]^-$. HRMS (ESI) *m/z* (-): calculated for $C_{17}H_{11}O_6$ $[M-H]^-$ 311.0550, found 311.0548.
